# Advances in Targeting the Epidermal Growth Factor Receptor Pathway by Synthetic Products and Its Regulation by Epigenetic Modulators as a Therapy for Glioblastoma

**DOI:** 10.3390/cells8040350

**Published:** 2019-04-12

**Authors:** Muhammad Nadeem Abbas, Saima Kausar, Feng Wang, Yongju Zhao, Hongjuan Cui

**Affiliations:** 1 State Key Laboratory of Silkworm Genome Biology, Southwest University, Chongqing 400715, China; abbasmndr@gmail.com (M.N.A.); drkausarsn@hotmail.com (S.K.); fengwang_swu@163.com (F.W.); 2 Engineering Research Center for Cancer Biomedical and Translational Medicine, Southwest University, Chongqing 400715, China; 3 Chongqing Engineering and Technology Research Center for Silk Biomaterials and Regenerative Medicine, Southwest University, Beibei, Chongqing 400715, China; 4 Cancer center, Medical Research Institute, Southwest University, Chongqing 400715, China; 5 College of Animal and Technology, Southwest University, Chongqing 400715, China; zyongju@163.com

**Keywords:** EGFR, glioblastoma, therapeutic targeting, epigenetic drugs, cell proliferation

## Abstract

Glioma is the most common primary tumor of the nervous system, and approximately 50% of patients exhibit the most aggressive form of the cancer, glioblastoma. The biological function of epidermal growth factor receptor (EGFR) in tumorigenesis and progression has been established in various types of cancers, since it is overexpressed, mutated, or dysregulated. Its overexpression has been shown to be associated with enhanced metastatic potential in glioblastoma, with EGFR at the top of a downstream signaling cascade that controls basic functional properties of glioblastoma cells such as survival, cell proliferation, and migration. Thus, EGFR is considered as an important therapeutic target in glioblastoma. Many anti-EGFR therapies have been investigated both in vivo and in vitro, making their way to clinical studies. However, in clinical trials, the potential efficacy of anti-EGFR therapies is low, primarily because of chemoresistance. Currently, a range of epigenetic drugs including histone deacetylase (HDAC) inhibitors, DNA methylation and histone inhibitors, microRNA, and different types of EGFR inhibitor molecules are being actively investigated in glioblastoma patients as therapeutic strategies. Here, we describe recent knowledge on the signaling pathways mediated by EGFR/EGFR variant III (EGFRvIII) with regard to current therapeutic strategies to target EGFR/EGFRvIII amplified glioblastoma.

## 1. Introduction

Glioma is the most common primary tumor of the nervous system, and approximately 50% of patients exhibit the most aggressive form of the cancer, glioblastoma [1,2]. A characteristic histopathologic hallmark of glioblastoma is marked cytologic heterogeneity of astrocytic cancer cells associated with necrosis and/or vascular endothelial proliferation [3,4]. Molecular and biological studies show that glioblastoma is the common endpoint in the progression of low-grade (grade 2) or anaplastic (grade 3) astrocytomas [5,6]. In the revised World Health Organization (WHO) classification of tumors of the central nervous system, glioblastoma is classified under the group of astrocytic tumors (grade 4), as opposed to its prior classification under embryonal tumors [7,8].

Epidermal growth factor receptor (EGFR) is a transmembrane protein involved in a broad range of developmental processes and human cancers, including glioblastoma, neck and head cancer, colorectal cancer, and non-small-cell lung cancer [9,10,11]. It has been shown that 40–50% of glioblastoma patients have dysregulated EGFR, and approximately half of these co-express the mutant receptor EGFR variant III (EGFRvIII) [12]. This mutant form is found in 20–30% of all glioblastoma patients. The EGFR family comprises four homologous members: ErbB1/EGFR/human epidermal growth factor receptor (HER)1, ErbB2/HER2, ErbB3/HER3, and ErbB4/HER4 [13,14]. All of the members share various common characteristics, such as a single transmembrane or membrane-spanning region, an extracellular domain (ECD) with two cysteine-rich regions, a juxtamembrane cytoplasmic domain, and an intracellular kinase domain with multiple C terminal tyrosine residues, which are phosphorylated on binding of ligand and activation of receptor [10,13]. The activated receptor subsequently initiates intracellular downstream signaling, such as the phosphatidylinositol 3-kinase (PI3K) and mitogen-activated protein kinase pathways [15]. Therefore, EGFR signaling influences different cellular processes, including metabolism, proliferation, and survival [16,17]. 

Besides EGFR overexpression and mutation, epigenetic mechanisms are increasingly known to be key contributing factors in the progression and development of glioblastoma [18,19]. Epigenetic modifications, specifically those linking to DNA modifications and associated histone proteins to modulate gene expression, are being exploited for therapeutic drug targeting and as biomarkers with prognostic significance. Epigenetic silencing of O-6 methylguanine-DNA methyltransferase through promoter hypermethylation has been shown to be related to remarkably longer survival [20,21].

Considerable differences appeared between EGFR abnormalities when data from clinical trials were combined with results from functional studies of tumorigenic EGFR signaling. Some EGFR abnormalities could potentially be treated with monotherapy, and those that are insensitive to such treatment could be targets for combination therapy in selected patient populations. Furthermore, epigenetic modifications such as histone acetylation and promoter methylation status are reversible and can be treated by synthetic drugs. Histone deacetylase (HDAC) inhibitors and DNA methyltransferase (DNMT) inhibitors have been tested in different cancers, but only HDAC inhibitors have entered clinical trials in glioblastoma. In the current review, we address the various mechanisms of tumorigenic EGFR signaling, focusing on the successes and limitations of EGFR inhibitors in the clinic, as well as different epigenetic regulators that not only modulate epigenetic modification but also affect EGFR signaling, and discuss recent knowledge that provides insight into the varied therapeutic effects of anti-EGFR therapy and epigenetic regulators.

## 2. Epidermal Growth Factor Receptor Family and Their Ligands

Epidermal growth factor receptor (EGFR, ErbB1) was the first member of the receptor tyrosine kinase family to be described. Later, three additional members of this family—ErbB2 (HER2), ErbB3 (HER3), and ErbB4 (HER4)—were discovered [22,23]. These are transmembrane receptors that show functional similarity and have similar conserved domains, e.g., a single hydrophobic transmembrane segment, an extracellular domain, and an intracellular one with protein kinase activity [24,25]. The extracellular ligand binding site of EGFR protein is made up of four domains: I, II, III, and IV. Domains II and IV combine with each other and inhibit the interaction of domains I and III to form the affinity binding site for ligand, hence the receptor activation is executed through domains I and III and other extracellular domains moderate the dimerization of the receptor and interactions with other membrane proteins [26,27,28]. 

Signaling from the receptors of the EGF family is regulated by a set of ligands (EGF-like ligands). To date, seven EGF-like ligands have been described, which can be categorized as high-affinity ligands of EGFR (transforming growth factor-alpha (TGF-α), betacellulin (BTC), epidermal growth factor (EGF), and heparin-binding growth factor (HB-EGF)) and low-affinity ligands of EGFR (epiregulin (EREG), amphiregulin (AREG), and epigen (EPGN)) [29,30]. These ligands form homo- or heterodimerization with the receptors of the EGFR family for receptor activation, thereby stimulating the intracellular domain with tyrosine kinase signaling to activate the signaling cascade. Without the interaction of EGFR receptor with EGF-like ligands, any activity from these kinase receptors is suppressed by accumulation of tyrosine phosphatase [30]. The interaction between ligands and EGFR receptors regulates different biological processes, particularly various kinds of progression and pathogenesis, by modulating the signaling pathways that control cellular growth and survival [31].

Enhanced EGFR abundance and signaling in glioblastoma can occur by different mechanisms, such as alterations in genetic information (amplification of receptor, mutation, and chromosomal translocation), angiogenesis, autocrine and paracrine signaling, and epithelial–mesenchymal transition [32,33].

## 3. Epidermal Growth Factor Receptor Amplification

In cells under normal physiological conditions, EGFR regulates different biological processes; however, any abnormality can lead to disease. The functions of different EGFR family members in physiologically normal and cancerous tissues have been summarized in many reviews [29,34,35]. For instance, overexpression and amplification of EGFR have been described in brain tumors, bladder cancer, lung cancer, and so on [36,37,38]. EGFR gene (chromosome 7) overexpression and amplification occur because of genetic alteration, which is associated with glioblastoma and reported in the 2016 WHO classification [39]. Many previous studies observed the overexpression of EGFR in primary glioblastoma. Yoon et al. [40] found EGFR amplification in primary glioblastoma (73.3%) and secondary glioblastoma (9.5%). Another study, which was performed on subgroupings (primary and secondary glioblastomas) described five cases out of 34 glioblastomas with EGFR amplification [41]. Similarly, Watanabe et al. [42] reported a 63% incidence of EGFR amplification in primary glioblastomas compared to only 10% in secondary glioblastomas. Recently, Thorne et al. [43] showed that EGFR is the most common form of alteration associated with glioblastoma by overexpression and amplification of this gene. They analyzed that approximately 40% of primary glioblastomas show alteration of the EGFR gene, while this pattern has rarely been identified in secondary glioblastomas. The authors further observed that all of the primary glioblastomas showed EGFR overexpression. However, approximately 70–90% of primary glioblastomas with EGFR overexpression demonstrated EGFR amplification.

## 4. Alteration of EGFR and Other Contributing Factors in Glioblastoma

In addition, glioblastoma with amplification of EGFR might describe additional mutations in the EGFR gene. This alteration includes in-frame deletion of exons 2–7 (loss of 841 base pairs or 267 amino acids of the full-length receptor, EGFRvIII) [44]. This altered EGFR glycoprotein lacks the cysteine-rich (CR1) and L1 subdomains of the ectodomain, and hence loses the ability to be activated by any EGF ligands. This mutant protein acquires the constitutive activation that facilitates and supports growth, and mitogenic signaling in the cell fosters malignancy [45]. This condition is present in approximately 50–60% of glioblastoma patients with EGFR amplification [43]. However, another study opposed this and demonstrated that improved survival of glioblastoma patients benefiting from metronomic temozolomide (TMZ)-based therapies and indicating an overexpression of EGFR is associated with an activated EGFR/PI3K/Akt pathway independent of the presence or absence of EGFRvIII [46]. It is therefore important to determine EGFR gene status for better classification of patients into groups included in clinical trials, but also for personalized therapies [47]. 

Besides alterations of EGFR and EGFRvIII, loss of heterozygosity (LOH) is also a contributing factor in the progression of glioblastoma. Alteration of LOH#10 is most frequent, as it occurs in 60–80% of glioblastomas. Moreover, in many cases of glioblastoma, the loss of one entire copy of chromosome 10 has been observed. The LOH occurs most often at 10p14-p15, 10q23-24, and 10q25-pter loci, indicating the existence of various tumor suppressor genes [48,49,50]. These studies suggest that LOH along with the other factors play a crucial role in the development of glioblastoma. 

Brennan et al. [51] determined the genomic alterations that most frequently occur in glioblastomas by analyzing more than 500 samples of the disease. They observed that besides the mutations in signature oncogenes of glioblastoma (e.g., EGFR and phosphatidylinositol 3-kinase (PI3K)), more than 40% of patients had mutations among the chromatin-modifier genes, which play a major role in chromatin organization in glioblastoma pathology [52,53]. Moreover, mutations in BRAF [54], various fibroblast growth factor receptor (FGFR1, FGFR2, and FGFR3) [55] also have potential impact on the pathogenesis of this disease.

Structural rearrangements, which contribute to the overall genome complexity, have also been found in glioblastoma patients. An example is the great frequency of structural variants on the q arm of chromosome 12, involving the cyclin-dependent kinase 4 (CDK4) and mouse double minute 3 (MDM2) genes, which may have some functional importance in this disease [51]. Additionally, EGFR fusion and deletion variants are largely distributed in glioblastoma patients. The development of a therapeutic strategy targeting mutated EGFR could have a major impact on survival and continues to be a topic of great interest [56]. Overall, approximately 148 different kinds of mutations have been suggested to be related to glioblastoma. Hence, developing therapeutic strategies targeting EGFR may be of benefit, if other contributing factors are also considered [57]. 

## 5. Targeting EGFR in Glioblastoma Using Drugs

**Figure 1:**
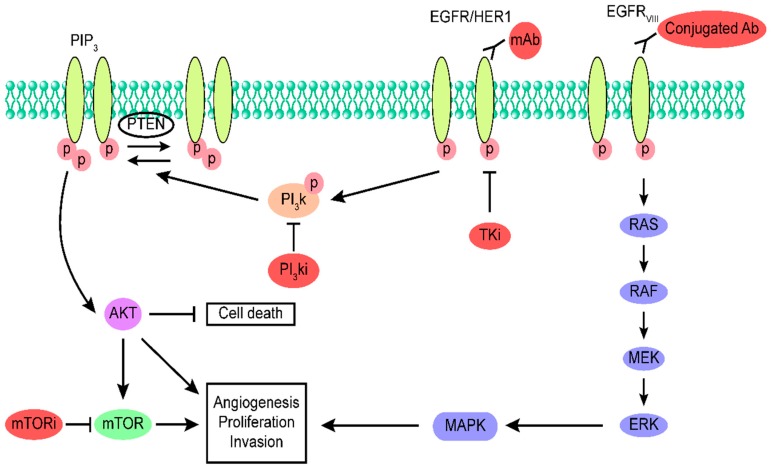
Epidermal growth factor receptor (EGFR)/EGFR variant III (EGFRvIII)-dependent biological signaling pathways. EGFR and EGFRvIII are able to induce signaling through canonical receptor tyrosine kinase (RTK) pathways comprising the RAS-RAF-MEK-ERK pathway and phosphatidylinositol 3-kinase (PI3K) signaling pathway.

The contribution of abnormal EGFR signaling to glioblastoma has led to the development of various therapies targeting the EGFR signaling pathway. These therapies include synthetic products, which are considered to be potential therapeutic options for glioblastoma. Some of these therapies, such as monoclonal antibodies, tyrosine kinase inhibitors, and vaccines, have already been approved by the US Food and Drug Administration for use in different types of cancers [58]. In this section, we discuss the existing information on chemical therapeutic options for glioblastoma (Figure 1).

### 5.1. Monoclonal Antibodies as Therapeutic Agents for Glioblastoma

Recent advancements in the field of EGFR-targeted therapies have shown that various types of inhibitors can be administered to cure brain cancers; for instance, tyrosine kinase has been suggested to provide benefit for some patients whose cancers have particular genetic aberrations. However, cancer patients with EGFR mutations usually gain resistance to tyrosine kinase inhibitors, reducing the average time to progression of disease to a few months [59].

To overcome this resistance, various strategies have been proposed, including monoclonal antibodies. Currently, patients are administered monoclonal antibodies, which directly bind to mutated EGFR [60]. These antibodies can be classified into blocking antibodies, which inhibit binding of ligand and can be receptor non-activating or activating and stimulate internalization or not. The blocking and internalized antibodies are considered ideal for suppressing receptor activity. To date, many antibodies have been developed against EGFR to treat glioblastoma patients. The EGF blocker cetuximab, first used for colorectal cancer, is an antibody that blocks binding of ligand without receptor activation [61]. Patel and co-workers [62] demonstrated that cetuximab can successfully bind to both EGFR and its variant EGFRvIII in U87MG cells. Further, cetuximab–EGFRvIII internalizes from the cell surface. This internalization then leads to a decrease in the phosphorylated form of the EGFRvIII receptor in transfected cells and a significant reduction in proliferation of cells. 

The phase II studies of the EGF blocker cetuximab showed limited effect in patients with recurrent glioblastoma [63] or radiotherapy [64] or in combination with irinotecan and bevacizumab [63]. Hence, cetuximab administration in glioblastoma was not clinically developed beyond phase II studies. However, some studies solved the delivery issue of this agent using direct intracranial infusion; the cetuximab successfully blocked glioblastoma cell invasion in a xenograft model, although this was found only in cells with high expression of EGFR and its variant EGFRvIII [65]. Likewise, a recent study developed another intracranial strategy to deliver the coding sequence for cetuximab directly to the central nervous system, and this strategy is also in preclinical trials. This study used an adeno-associated virus serotype rh.10 gene transfer vector to deliver the corresponding antibody sequence directly to the central nervous system by intracerebral injection so that the anti-EGFR antibody would be generated through the transduced cells to enhance the cetuximab concentration [66]. Concurrently, in a clinical trial Chakraborty and co-authors [67] studied the safety of osmotic blood–brain barrier opening with an infusion of mannitol followed by intra-arterial cetuximab infusion. Further cetuximab-based progress comprises EGFR boronation with anti- cetuximab antibodies [68] or later the development of bioconjugates with platinum derivatives [69]. The boronated compounds, which were chemically linked to cetuximab, were utilized to study boron neutron capture therapy. These compounds were largely found to be ineffective in glioblastoma. Similarly, the cetuximab-based bioconjugates utilized with platinum derivatives also had an effect in terms of their efficacy. However, it was suggested that treatment of glioblastoma patients with anti-EGFR antibody increases the radiation effect [70], but this is expected to be most likely an influence on the antibody-exposed vasculature and thus an indirect cancer cell EGFR-independent influence. Direct use of radiolabeled monoclonal antibodies against EGFR has also been disappointing [71]. 

Nimotuzumab is another monoclonal antibody with different characteristics. Nimotuzumab has lower binding affinity compared to cetuximab, and hence it more specifically binds to the extracellular domain of EGFR, thereby blocking the ligand binding and receptor activation [72]. Further, it showed potential efficacy in multicenter trials against glioblastoma [73] and against high-grade glioma in phase II trials [74]. In a multicenter, open label, prospective, two-armed, randomized phase III trial to examine the potential efficacy of nimotuzumab in combination with radiation therapy for newly diagnosed glioblastoma patients, it showed high efficacy in these patients [73,75]. Unfortunately, the study tested multiple parameters and its inclusion criteria were very broad, so it failed to prove efficacy in this subgroup [76]. Further, nimotuzumab use in combination with radiation and vinorelbine in patients with newly diagnosed diffuse intrinsic pontine glioma showed promising efficacy and an increased median survival of 15 months [77]. 

### 5.2. Tyrosine Kinase Inhibitors as Therapeutic Agents for Glioblastoma

Multiple tyrosine kinase inhibitors (e.g., gefitinib and erlotinib) have been developed to suppress EGFR signaling. Gefitinib and erlotinib are low-molecular-weight, reversible, oral tyrosine kinase inhibitors associated with EGFR signaling [78]. These inhibitors compete with adenosine triphosphate and reversibly bind to the intracellular tyrosine kinase domain of EGFR or EGFRvIII, suppressing autophosphorylation of the receptor and further downstream signaling [12]. Gefitinib and erlotinib were shown to have anti-glioblastoma activity in preclinical studies [79]. For instance, gefitinib and erlotinib caused the repression of cellular viability of glioblastoma-derived tumor-initiating cell lines at particular concentrations. The suppression of cellular viability is associated with reduced EGF-induced phosphorylation of EGFR/HER1 with the consequent suppression of both Akt and ERK1/2 activation, thereby inhibiting the mitogen-activated protein kinase (MAPK) signaling pathway [80]. Furthermore, it has already been shown that the extent of erlotinib-mediated suppression of anchorage-independent growth of glioblastoma-derived cell lines is inversely correlated with the cellular capability to induce EGFR/HER1 messenger ribonucleic acid (mRNA), highlighting the important role of EGFR/HER1 in the pathogenesis of glioblastoma [81].

In subsequent phase I studies, erlotinib displayed a reasonable safety profile and was usually well tolerated. Additionally, enzyme-inducing antiepileptic drugs (EIAEDs) were found to enhance erlotinib metabolism, requiring dose modification or a change in the antiepileptic drug regimen [82]. Later, in a phase II study of 43 patients with non-progressive glioblastoma and 53 patients with recurrent glioblastoma, the application of erlotinib after radiotherapy demonstrated median progression-free survival at only 2 months and median overall survival at 6 months for patients with recurrent glioblastoma, while median overall survival was 14 months and 12-month overall survival was 57% for patients with non-progressive glioblastoma [83]. Hence, this phase II study did not show remarkable improvement in patients with non-progressive or recurrent glioblastoma following treatment with erlotinib. However, another study reported that median overall survival and 6-month progression-free survival of recurrent glioblastoma patients (*n* = 48) after erlotinib exposure exceeded historical values for cancer patients receiving chemotherapy for recurrent glioblastoma [84]. However, this study was stopped due to an inadequate number of responses following a planned interim analysis, and a control group was not included. Another study reported that in a randomized controlled phase II trial, only 11.4% of patients (*n* = 54) with recurrent glioblastoma who were given erlotinib remained free of development after 6 months compared to the control group (24.1% of patients), who received either bis-chloroethylnitrosourea or temozolomide [85]. Moreover, median overall survival was shown to be similar across the treatment groups (7.3 months for the BCNU/temozolomide group versus 7 months for the erlotinib group).

The first phase II study of gefitinib treatment was performed in 2004, which suggests that this drug is well tolerated and has activity in patients with recurrent glioblastoma. This study was done with a total of 53 patients, demonstrating 6-month event-free survival in 13% of patients. The median event-free survival time and median overall survival time from treatment initiation were 8.1 and 39.4 weeks, respectively [86]. 

### 5.3. mTOR Inhibitors as Therapeutic Agents for Glioblastoma

EGFR impairment and variation in phosphatase and tensin homolog (PTEN) gene expression cause enhanced activity of the PI3K-Akt-mTOR signaling pathway [87]. The mTOR complex has a key biological role in the regulation of metabolism, protein synthesis, and angiogenesis. Any functional irregularity in mTOR has been shown to be involved in the development of glioblastoma, and thus it has been suggested that mTOR signaling pathway inhibition may have therapeutic value in this disease [88,89]. Numerous studies have suggested that mTOR inhibitors are effective therapeutic agents for the treatment of different types of cancers [58]. mTOR inhibitors such as rapamycin and its analogs (everolimus (RAD001), deforolimus (AP23573), and temsirolimus (CCI-779)) suppress cellular growth and proliferation and are considered to be effective for glioblastoma treatment [90,91]. These therapeutic agents form a complex after binding with FK506 binding protein 12, which interacts with mTOR, thereby inhibiting the key signaling pathways and causing cell cycle arrest at G1. Based on the solubility of mTOR inhibitors, they are administered either orally or intravenously. These agents have the ability to penetrate the blood–brain tumor barrier. For instance, patients treated with the mTOR inhibitors temsirolimus and sirolimus showed a measurable concentration of temsirolimus and sirolimus in tumor tissue. Further, this study showed that tumor tissue/whole blood concentration ratios of temsirolimus and sirolimus were 1.43 and 0.84, respectively, in the studied patients [92]. Moreover, recent studies suggest that combined administration of EGFR–mTOR inhibitors represses growth and proliferation of tumor cells and suppresses the PI3K signaling pathway in glioblastoma. Additionally, this combination therapy induces cell death in PTEN-deficient tumor cells [93]. Later, Tanaka and coworkers [94] reported that mTOR-targeted therapies influenced the use of glutamine and induced pathways by providing glutamine carbon to the citric acid cycle, enhancing glutaminase expression. Targeting glutaminase as a therapeutic strategy may be a rational approach in the future for mTOR-targeted combination therapy, and similarly, the assembly of EGFR and EGFRvIII for the induction of signal transducer and activator of transcription (STAT) signaling. Combination therapy that blocks STAT activation has been suggested to remove nontarget impacts that underlie mTOR kinase inhibitor leading to cell apoptosis. Blocking of STAT signaling using a combination of EGFR and Janus kinase inhibitors has been associated with apoptosis of cells in glioblastomas. The combined use of approved Janus kinase and EGFR inhibitors could be a new strategy for the treatment of cancer patients [95].

### 5.4. PI3K Inhibitors as Therapeutic Agents for Glioblastoma

Phosphoinositide 3 kinase has been shown to be a major modulator of diverse cellular functions such as cell growth and proliferation, protein synthesis, cell cycle regulation, glucose metabolism, survival, differentiation, and motility [96,97,98]. Phosphoinositide 3 kinase signaling pathway activation has been demonstrated in different types of human cancers, including glioblastoma, because of gain-of-function mutations in PIK3CA or loss of PTEN [99]. Recent studies have shown that components of the phosphoinositide 3 kinase signaling pathway are often targeted by somatic or germline mutations in a broad spectrum of cancers including glioblastoma [100]. Phosphoinositide 3 kinase inhibitors such as wortmannin and LY294002 are broadly used in in vitro experiments, while pharmacological characteristics such as instability and insolubility in vivo limit their clinical application [101]. However, several drugs with improved pharmaceutical characteristics to target phosphoinositide 3 kinase have been developed for clinical trials [102]. Phase I clinical studies with these therapeutic agents are in development in different types of cancers. XL765, a dual mTOR and PI3K oral inhibitor, is the first member of this class to be examined in glioblastoma patients; a phase I trial combining radiation therapy (RT)/TMZ with XL765 in patients with recurrent gliomas is under way.

## 6. Epigenetics in Glioblastoma

Epigenetics are defined as the heritable variations in the cellular DNA that do not involve variations in the sequence of DNA. Epigenetics modulate the architecture of chromosomes, control gene expression, and ultimately govern heritable phenotypes from genotypes. Modifications in epigenetics such as DNA methylation, nucleosome remodeling, histone modification, and noncoding RNAs greatly influence vital biological processes and are involved in the initiation and progression of tumors [103,104]. Six epigenetics-based glioblastoma subgroups have been identified, which exhibit characteristic global DNA methylation patterns possessing discrete hotspot mutations, alterations in DNA copy numbers, and transcriptomic patterns [105]. Loss of heterozygosity of chromosome 10q is the most common epigenetic modification in glioblastoma [106]. Many cancer mutations cause modifications in DNA methylation patterns, nucleosome positioning, noncoding RNA changes, and histone modifications, which interrupt key biological signaling pathways [107]. These epigenetic changes can influence the balance of various proteins in a cell; for example, changes in O6-methylguanine-DNA-methyltransferase (MGMT) methylation patterns might cause decreases in PMS2, MSH2, and MSH6, proteins in glioblastoma [108]. Furthermore, many tumor suppressors, cell-cycle regulators, and DNA repair genes are also affected by epigenetic modifications in glioblastoma [109,110].

### 6.1. Modulation of EGFR-Dependent Signaling Pathways in the Context of Epigenetic Drugs in Glioblastoma

**Figure 2:**
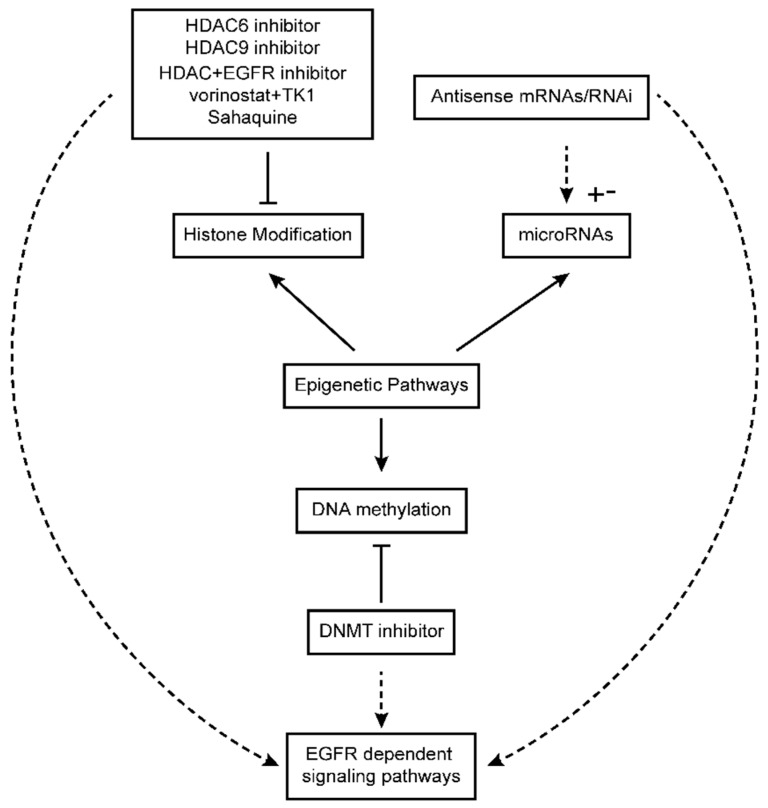
Effect of epigenetic drugs on epigenetic pathways and EGFR-dependent biological signaling pathways. HDAC: histone deacetylase; DNMT: DNA methyltransferase.

Despite great advancements over the past decade in targeting EGFR in glioblastoma using synthetic products, there is still considerable room for further progress. It has been shown that most glioblastoma patients show little or no response to therapy using EGFR inhibitors, which suggests an intrinsic resistance against these inhibitors. Even in those patients who do achieve an obvious cancer response to clinically used EGFR inhibitors, most will ultimately display progression and development of this disease, which implies developed resistance in these patients. Epigenetic modification is also a contributing factor in the development of resistance against EGFR inhibitors in cancer patients. Thus, alternative approaches such as epigenetic drugs are considered good options to overcome resistance problems in patients. Many epigenetic drugs are undergoing clinical trials and some of them have already been approved for glioblastoma treatment by the European Medicines Agency and the Food and Drug Administration [111]. Targeting epigenetic regulators in glioblastoma can also modulate the activity of EGFR and its associated pathways (Figure 2). Treatment with epigenetic regulators or in combination with EGFR inhibitors represents a new hope for treatment of glioblastoma [112,113,114,115]. 

### 6.2. DNA Methylation Inhibitors in Glioblastoma Treatment and Their Impact on EGFR-Dependent Pathways

DNA methylation is the biological process by which methyl groups are added to DNA. It was one of the earliest discovered epigenetic alteration pathways. In DNA, there are four possible sites for DNA methylation: the N-4 position of cytosine, the C-5 position of cytosine, the N-6 position of adenine, and the N-7 position of guanine [116,117]. In mammalian cells, DNA methylation occurs mostly in the cytosine of 5′-CpG-3′ to generate 5-methylcytosine, which is catalyzed by DNMT enzymes [118,119,120]. Several studies have demonstrated that DNA methylation can contribute to altered DNA conformation, DNA stability, chromatin structure, and interactions between DNA and proteins, and can also regulate gene expression. Furthermore, DNA methylation has become an important field in epigenetic genomics and epigenetics studies due to the close relationship between DNA methylation and the development of cancer diseases, particularly the transcriptional inactivation of tumor suppressor genes caused by the methylation of CpG island content [121,122,123].

Metastasis suppressor protein 1 (MTSS1) is generally expressed in tissues such as thymus, spleen, uterus, prostate, colon, and peripheral blood, but is absent or has low expression in various cancers, including gastric [124], bladder [125], breast [126], and colorectal [127] malignancies, where its decreased transcription correlates with poor patient survival. In basal cell carcinomas, MTSS1 has also been identified as a sonic hedgehog responsive gene, potentiating Gli-dependent expression [128]. Additionally, MTSS1 regulates the EGFR signaling pathway [129]. MTSS1 DNA methylation and transcriptional silencing has been reported in bladder cancer cells, with detection of a promoter activity region 276 bp upstream of the metastasis suppressor protein 1 (MTSS1) gene within a CpG island [130]. Furthermore, Fan and co-workers demonstrated a DNA methylation independent silencing mechanism by DNA methyltransferase 3B (DNMT3B) in hepatocellular carcinomas [131]. Most recently, Schemionek and colleagues demonstrated MTSS1 as an epigenetic regulated tumor suppressor in chronic myeloid leukemia [132]. Demethylation of DNA by 5-aza-2′-deoxycytidine in glioma cells enhanced the mRNA expression of MTSS1, which increased the overall survival of glioblastoma patients [133]. 

Amplification and rearrangement of the EGFR gene have frequently been reported in glioblastoma. The most common variant is EGFR variant III (EGFRvIII), which is a key marker for tumor-initiating cells. In primary glioblastoma, expression of EGFRvIII is focal and sporadic [134,135]. Del Vecchio and co-workers [136] described that EGFRvIII-negative short-term cells maintain levels of amplification comparable to EGFRvIII-positive cells and re-expressed EGFRvIII independent of 5-aza-2′-deoxycytidine stimulation over the course of several weeks in in vitro analysis. They further suggested the re-expression of EGFRvIII with time may be due to the gradual loss of amplicons, as the cells differentiate and therefore are no longer pressured to maintain the presence of amplicons [136].

### 6.3. Histone Deacetylase Inhibitors in Glioblastoma Therapy and Their Influence on EGFR Signaling Pathway

Histone deacetylases (HDACs) are an important group of intracellular enzymes, which have been demonstrated to be involved in epigenetic modifications in the human body. HDACs modulate chromatin remodeling by stimulating the removal of acetyl residues from both nonhistone and histone proteins, thereby enhancing the condensation status of chromatin and suppressing the transcription of genes [137,138]. HDAC inhibitors are an important group of antitumor agents [139] and are used to inhibit HDAC proteins in glioblastoma patients, thereby increasing gene transcription, including loci that became epigenetically silenced during the development and progression of tumors. In tumor cells, this process leads to the stimulation of differentiation and proapoptotic programs, which in turn mediate the antitumor effects of HDAC inhibitors [140,141]. Growing evidence suggests that HDAC inhibitors remarkably decrease the transcription of genes, particularly those that have high copy numbers, such as EGFR, in different types of cancers, including glioblastoma, breast cancer, and so on [142,143]. Thus, HDAC inhibitors are a highly important group of therapeutic agents that are being evaluated for treating various types of cancers, including glioblastoma.

HDAC6 protein belongs to the class IIb HDAC family, which deacetylates different types of substrates such as Hsp90, α-tubulin, and cortactin in both nucleus and cytoplasm [144]. In addition, this protein also plays a key biological role in the regulation of tumor-related signaling pathways, especially the EGFR pathway [145]. A growing number of studies have reported abnormal expression profiles of HDAC6 in different types of tumors such as glioblastoma [146], oral squamous cell carcinoma [147], breast cancer [148], ovarian cancer [149], and model organisms. Recent studies have shown that HDAC6 protein enhances cell proliferation and imparts temozolomide resistance in glioblastoma [146,150]. Further, it has been shown that HDAC6 protein is upregulated in glioblastoma tissues and its cell lines. This upregulation of HDAC6 protein facilitates the cell proliferation and spheroid formation of glioblastoma cells and renders them resistant to temozolomide. Conversely, inactivation or suppression of HDAC6 induces apoptosis, inhibits cell proliferation, delays spheroid formation, and renders glioblastoma cells more sensitive to temozolomide. Furthermore, resistance of temozolomide is related to EGFR activation and increased HDAC6 expression. The HDAC6 inhibitors CAY10603, ACY-1215, and tubastatin A abrogate resistance of temozolomide by reducing and inactivating EGFR protein expression. These observations indicate that the suppression of HDAC6 is an effective and novel therapeutic approach for glioblastoma treatment and overcoming resistance to EGFR inhibitors such as temozolomide [146,150,151]. 

Similarly, it has been shown that the transcription level of HDAC9 (belonging to the class IIa HDAC family) is remarkably increased in different types of cancers, including glioblastoma [152], medulloblastoma [153], cervical cancer [154], and acute lymphoblastic leukemia [155]. HDAC9 protein is generally upregulated in glioblastoma patients who have a poor prognosis. This protein promotes cell proliferation in glioblastoma and tumor formation through induction of the transcription co-activator with a PDZ-binding motif (TAZ)-mediated EGFR signaling pathway. Further, it can directly interact with TAZ, an oncogene and important downstream effector of the Hippo pathway. Conversely, suppression of HDAC9 decreases the transcription of TAZ [152,156]. A remarkable effort is ongoing to find new inhibitor molecules targeting class IIa HDAC proteins such as HDAC9. However, so far, no HDAC9 protein-specific inhibitors have been found. Still, these studies shed light on new evidence of a promising target for glioblastoma treatment. The HDAC inhibitor molecule CUDC-101 simultaneously targets human epidermal growth factor receptor 2 (HER2) and EGFR. This molecule showed potent proapoptotic and antiproliferative effects against different cancer cells, including glioblastoma [157].

Another highly effective combination of HDAC inhibitors is with erlotinib (an EGFR tyrosine kinase inhibitor). The combination of HDAC inhibitor + EGFR inhibitor can prevent the development of resistance in glioblastoma cells [158]. Further, this study suggested that treatment with CUDC-101 (a multitargeted EGFR/HDAC inhibitor) can also reduce the development of resistance in these cells. Mechanistically, this study found an HDAC inhibitor-dependent decrease in expression of EGFR/EGFRvIII protein underlying the antiproliferative effects of the HDAC inhibitors. Thus, HDAC inhibitor combined with erlotinib is a promising treatment option for newly diagnosed, treatment-naïve tumors irrespective of their EGFR status, and for treatment-refractory EGFR-overexpressing glioblastoma. Overall, combining EGFR targeted therapy with HDAC inhibition reduces the development of resistance in glioblastoma cells in vitro by preventing compensatory overexpression of wild-type EGFR/EGFRvIII and represses wtEGFR/EGFRvIII in cells that have attained resistance to targeted therapy by increased expression of these oncogenes, thereby resensitizing the cells to targeted therapy. Moreover, these findings are transferable to nonamplified glioblastoma cells, for which a combination of HDAC inhibitor with erlotinib also additively inhibits proliferation. However, the clinical usability of a combined EGFR/HDAC-targeted approach might be limited due the fact that current clinical trials combining vorinostat, a class I/II HDAC inhibitor, with erlotinib, including one trial on recurrent glioblastoma (#NCT01110876), had to be terminated because of the unexpected toxicity of this combination of drugs [159].

Furthermore, combining the HDAC inhibitor vorinostat with a multitargeted tyrosine kinase inhibitor (TKI), vandetanib, can prevent EGFR, vascular EGFR (VEGFR), and Ret-dependent signaling, thereby inhibiting growth, preventing Akt signaling, and stimulating apoptosis in glioblastoma cells [160]. Marino et al. [161] used a combination of 4-phenylbutyrate with vandetanib or gefitinib and observed enhanced apoptosis and decreased clonogenic survival in glioblastoma cells. Adding the EGFR TKI erlotinib to vorinostat is an effective strategy to treat glioblastoma cells. This combination enhanced the antiproliferative effect in the treated cells, which was associated with activation of migration and invasion inhibitory protein (MIIP) and decreased transcription of platelet-derived growth factor receptor A [162,163]. 

The development of hybrid compounds is one of the most active areas in glioblastoma therapeutics. Hybrid molecules can have multiple targets, decreasing the risk of drug resistance, lowering effective doses, and reducing side effects [164,165]. A recent preclinical study used a novel hybrid molecule, sahaquine (*N*-hydroxy-*N*′-{4-[(6-methoxyquinolin-8-yl)amino]pentyl}pentanediamide) for glioblastoma treatment. Sahaquine contains hydroxamic acid and primaquine linked by dicarboxylic acid [166]. This hybrid molecule can selectively inhibit HDAC6 protein at low concentration, even at nanomolar concentration, without markedly reducing class I HDACs. HDAC6 inhibition leads to remarkable acetylation of α-tubulin, thereby impairing cytoskeletal organization in glioblastoma cells. Sahaquine suppresses P-glycoprotein activity, which contributes to glioblastoma drug resistance. Further, this study proposed a mechanism of action indicating that sahaquine reduces HDAC6 protein as well as EGFR and its downstream kinase activity [167]. This study showed that sahaquine is an effective therapeutic agent that can affect multiple cellular factors and signaling pathways that are crucial for glioblastoma treatment. Sahaquine’s effects can be further enhanced by administering this hybrid molecule in combination with temozolomide, quercetin, or buthionine sulfoximine. The investigation was aimed at determining the effect of sahaquine in combination with other synthetic or natural drugs in patient-derived organoids, and highlights new evidence of a promising therapeutic agent for glioblastoma treatment. 

### 6.4. MicroRNAs in Glioblastoma Therapy and Their Impact on EGFR Signaling Pathway

MicroRNAs are short noncoding intracellular RNAs that are involved in the regulation of posttranscriptional expression of genes in both animals and plants [168]. A total of 2588 mature and 1881 precursor miRNA sequences have been described as associated with humans, and a list of these miRNAs is available on miRBase (http://www.mirbase.org/cgi-bin/browse.pl?org=hsa). These miRNAs can regulate the transcription of different mRNAs by targeting complementary regions of the 3′-untranslated region (3′-UTR) of the mRNA, preventing posttranscriptional gene expression or degrading mRNA and ultimately decreasing protein levels [169]. Several studies have begun to map out the transcription patterns and biological roles of miRNAs in glioblastoma, aiming to gain new insight that can be utilized to combat this insidious disease. Moller and co-workers [170] surveyed the glioblastoma-associated miRNAs and found 253 overexpressed miRNAs, 95 of them down-modulated, whereas some miRNAs (*n* = 17) remain controversial as to whether they increase or decrease in glioblastoma cells. The genes that are regulated by miRNAs are involved in various biological pathways such as cell proliferation, autophagy, resistance to apoptosis, drug resistance, and so on. 

It has been shown that many miRNAs functions as tumor suppressors. These miRNAs, which interfere with the histone methyltransferase enhancer of zeste homolog 2 (EZH2), are believed to have anticancer properties, especially let-7, which represses oncogenes such as K-RAS and MYC [171,172] and is capable of preventing cell proliferation in glioblastoma [173]. miR-128 is another example of a miRNA that acts as a tumor suppressor in glioblastoma. This is an antiproliferative miRNA that interferes with biological pathways that target genes involved in glioblastoma pathogenesis, including EGFR and platelet-derived growth factor receptor alpha (PDGFRA) [174], E2F3a [175], and WEE1 [176]. miR-21 is one of the most frequently overexpressed miRNAs in glioblastoma cells. miR-21 is a PTEN regulator, and its suppression by specific antisense oligonucleotide can induce apoptosis and prevent cell proliferation in glioblastoma cells. Moreover, antisense-miR-21 can also suppress EGFR, Akt, cyclin D, and Bcl-2 in a xenograft model [177]. miR-299-5p is generally upregulated in glioblastoma, and its depletion can reduce the activity of the EGFR-related signaling pathway (MAPK/ERK). Further, this miRNA suppression increases the sensitivity of glioblastoma cells to temozolomide in both in vitro and in vivo analysis [178]. 

Some miRNA expression is decreased during the progression of glioblastoma, which targets EGFR transcripts to decrease the total number of receptors made by targeting NHE9. It routes the few EGFRs made away from the plasma membrane to dampen oncogenic signaling. Downregulation of NHE9 expression by miR-135a acidifies sorting endosomes, limiting EGFR traffic to the glioblastoma cell membrane [179]. miR-7 is a common regulator of the PI3K/Akt and Raf/MEK/ERK pathways, both of which are launched by EGFR, and is a potential glioblastoma suppressor that acts by targeting multiple oncogenes related to the downstream pathway of EGFR. Liu et al. [180] observed that the forced expression of miR-7 can suppress transcription of PI3K, phosphorylated MEK 1/2, phosphorylated Akt, Raf-1, and cyclin D1, and can decrease expression of EGFR and vice versa. Additionally, transient expression of miR-7 in glioblastoma cells strongly inhibited glioblastoma xenograft growth in vivo. miR-7 transfection in glioblastoma cells leads to repressed invasiveness, enhanced apoptosis, and negatively regulated EGFR and Akt pathways, fulfilling the basic requirements of a tumor suppressor [181,182,183,184]. Lentiviral inhibition of miR-566 suppresses tumor progression in glioblastoma cells by controlling apoptosis and cell proliferation. This miRNA can positively regulate the EGFR signaling pathway, and its inhibition sensitizes glioblastoma cells to anti-EGFR therapy [185]. A recent study described the association between miRNA-200c and EGFR. miRNA-200c is downregulated and ZEB1 is overexpressed when tumors have high levels of EGFR amplification. Thus, regulating miRNA-200c expression may be helpful in glioblastoma treatment [186].

miRNA expression is altered in glioblastoma, and it is now possible to manipulate the expression of miRNA by administering miRNAs, similar to the use of antisense mRNA and RNA interreference (RNAi) [187]. Artificial antisense miRNAs could be produced and used to block their targeted oncomiRs associated with EGFR to inhibit the progression of glioblastoma. This information will be helpful to develop epigenetic drugs to manipulate glioblastoma-associated miRNAs, which could have a bright future and become a novel therapeutic agent for glioblastoma treatment. 

## 7. Conclusions

Epidermal growth factor receptor (EGFR) is a key regulator of cell survival responses that uses the network of downstream signaling events leading to proliferation and growth of a cell. There has been an explosion of knowledge in the past decade in the understanding of the biological functions of EGFR and its associated pathways in glioblastoma. Overexpression of EGFR and EGFRvIII is frequently observed in glioblastoma, and is a key contributing factor in the progression of this disease. Glioblastoma, the most common malignant brain tumor in adults, remains incurable, with a bleak median survival. Targeting EGFR for glioblastoma treatment is an attractive therapeutic option. However, the development of resistance against EGFR inhibitors in glioblastoma patients stimulated the development of multitargeted drugs or epigenetic drugs or the use of a combination of drugs for glioblastoma treatment. Epigenetic modifications are also closely related to glioblastoma proliferation, invasion, metastasis, and prognosis. These include DNA methylation, histone modification, chromatin remodeling, and abnormal microRNA. The DNA methylation level of the gene promoters can be taken as a guide for glioblastoma diagnosis; also related to the prognosis of this disease [188], miRNAs are involved in many crucial biological functions in its progression and development [189]. The enzymes that control histone alterations are good candidates for the diagnosis of glioblastoma, and histone inhibitors are good candidate drugs for glioblastoma treatment. Many of these therapeutic agents also play critical biological roles in the regulation of EGFR [190,191]. Furthermore, these epigenetic phenomena might also assist in the monitoring of high-risk groups and assessment of tumor risk, judgment of cancer recurrence, prediction of cancer treatment efficacy, and development of particular new target synthetic drugs. Based on our review, epigenetic drugs such as HDAC inhibitors can be taken alone or combined with EGFR inhibitors or other drugs. The development of hybrid molecules and miRNA inhibitors is also particularly interesting and a novel direction for further treatment of glioblastoma. Because of the complicated pathogenesis of glioblastoma, epigenetic applications for clinical treatment are still scarce. In glioblastoma, exploring more effective therapeutic targets, developing new targeted synthetic drugs, upgrading the efficacy of drugs that are currently used in clinical research, and decreasing the side effects of clinically used drugs are the major problems that we face and need to solve in clinical treatment.
